# Accelerating Community Engagement: Measuring Results

**DOI:** 10.3389/ijph.2025.1608499

**Published:** 2025-12-16

**Authors:** Cyril Pervilhac, Akarsh Venkatasubramanian, Winnie Mpanju-Shumbusho, Luc Barriere-Constantin

**Affiliations:** 1 World Health Organization (retired) Technical Officer, Geneva, Switzerland; 2 Engineering for Global Health, Geneva, Switzerland; 3 World Health Organization, Geneva, Switzerland; 4 The Constellation, Dardagny, Switzerland

**Keywords:** qualitative methods, indicators, community engagement, monitoring and evaluation frameworks, international agencies

## Abstract

**Objectives:**

Community engagement (CE) is vital for Primary Health Care (PHC) and for achieving the UN Sustainable Development Goals (SDGs) by 2030. However, large public health programs often prioritize measuring outcomes and impact over assessing CE itself. This paper proposes a comprehensive, community-centered Monitoring and Evaluation (M&E) framework with relevant indicators to strengthen CE assessment.

**Methods:**

We reviewed international M&E frameworks and their applications from both public health and community perspectives. Our analysis drew on guidelines from international organizations and 10 years of project data from seven countries.

**Results:**

Findings underscore the need to bridge program-level and community-level indicators while aligning recent M&E guidance with CE frameworks from international agencies. Accordingly, we present a revised, comprehensive, community-centered M&E framework, along with supporting actions for its implementation—such as contextual adaptation, research, digital innovation, resource mobilization, and recommended policy measures.

**Conclusion:**

As the 2030 targets approach, strengthening normative and policy guidance on CE is essential to accelerate progress toward the SDGs. This publication reinforces CE’s central role in PHC, Universal Health Coverage, and sustainable development strategies.

## Introduction

In 2019, the World Health Assembly reaffirmed Primary Health Care (PHC) as the foundation for Universal Health Coverage (UHC), adopting a resolution on community health worker programs [1]. This commitment reinforced PHC within the broader goals of UHC and Sustainable Development Goal 3 (SDG 3: Good Health and Wellbeing) [2, 3]. In response, countries have adopted international Monitoring and Evaluation (M&E) frameworks, often with the support of global agencies. These indicators track national progress but primarily serve government priorities and are less adaptable to civil society needs.

Over the past two decades, Community Engagement (CE) has become a central pillar of national health strategies [4–8]. The World Health Organization (WHO) recognizes CE as a core component of PHC, particularly in the response to HIV/AIDS [9]. The 2023 World AIDS Day theme, “Let Communities Lead,” underscored the importance of M&E in supporting community leadership [10]. Empirical evidence consistently demonstrates that effective CE, supported by appropriate measurement tools, is essential to ensure that PHC systems remain responsive to community needs and priorities [11].

Since the 2000s, CE has been instrumental in scaling up vaccination and disease control programs targeting infectious diseases—including HIV/AIDS, tuberculosis, and malaria—as well as non-communicable and neglected tropical diseases [12, 13]. The Ebola epidemic and the COVID-19 pandemic further highlighted CE’s central role in health response and monitoring, with CE embedded in WHO Africa’s COVID-19 Strategic Response Plan and integrated into M&E frameworks across Europe, Asia, Africa, and the Americas [14–18].

Strengthening CE as a foundational pillar of PHC can enhance global health outcomes and advance sustainable development. However, existing M&E frameworks often overlook key CE principles such as community-led evaluation, participatory learning, and sustainability. While standardized outcome measures are critical for data quality, they frequently fail to capture CE’s nuanced and context-specific outcomes [19, 20]. A more comprehensive M&E approach is therefore required—one that supports community-led responses while maintaining rigorous assessment standards. Integrating CE indicators into standardized program indicator categories, complemented by community-based metrics, could help achieve this goal.

The main purpose of this paper is to highlight the need for national and global strategies that effectively engage communities in measuring CE activities and results within specific project implementations. Findings are based on a literature review of international M&E frameworks spanning over four decades, an analysis of community indicators used in Asian and African contexts, and lessons learned from an NGO with more than 20 years of experience in community-based interventions [21].

Accordingly, we propose a comprehensive, community-centered M&E framework aligned with current guidelines and informed by qualitative indicator examples. The framework is designed for application across diverse public health programs. Drawing on recent NGO experiences and country case studies, we discuss how it can be developed and adapted to address major challenges—including research gaps, digital solutions, and resource mobilization—and conclude with specific recommendations for key audiences.

## Methods

Our approach involved three steps: 1) reviewing existing monitoring and evaluation (M&E) frameworks, primarily in HIV/AIDS; 2) analyzing a decade of community engagement (CE) data from an international NGO across multiple countries; and 3) developing an updated M&E framework to strengthen CE indicator measurement through a multidisciplinary approach addressing cross-cutting issues. We built on recent implementation science recommendations emphasizing practical utility and impactful outcomes that benefit communities according to their own priorities, with researchers and communities jointly leading research and interpretation of findings [8]. The review was further supported by the authors’ hands-on expertise in designing international M&E frameworks, facilitating community health programs, and contributing to global and local reporting.

### Chronological Overview

We examined the evolution of community-led actions and outcome measurement since the Alma-Ata Declaration, identifying strengths, limitations, and opportunities within existing M&E frameworks. A literature review and critical synthesis of international M&E frameworks were conducted from a CE perspective, focusing primarily on HIV/AIDS but also including other infectious diseases. The review incorporated both published and grey literature from agencies such as UNICEF, WHO, UNAIDS, and the Global Fund (GF) [4, 22–24]. Our retrospective analysis (2000–2023) identified gaps in CE measurement but also highlighted recent and promising CE framework developments that informed the creation of our updated M&E framework.

### Case Studies

We analyzed 10 years (2012–2022) of CE experiences from The Constellation, an international NGO, across multi-country projects addressing infectious and chronic diseases, as well as broader development and primary health care (PHC) issues in seven countries: Algeria, Botswana, the Democratic Republic of the Congo, Ghana, Guinea, Liberia, and India. To integrate national and community perspectives, country case studies were conducted throughout project cycles using stakeholder mapping, participatory evaluation, and qualitative methods such as unstructured interviews and focus groups. We reviewed final evaluation reports and addressed missing data by sending questionnaires to key informants (KI), focusing on methodological challenges and findings related to evaluation design, qualitative methods, and outcome measurement. A comparative summary table (Supplementary Material 1: Summary of The Constellation Projects in 7 Countries) synthesizes these findings. This process enabled identification of the tools used for data collection and the indicators most commonly applied to measure CE.

### Comprehensive Community-Centered M&E Framework

**FIGURE 1:**
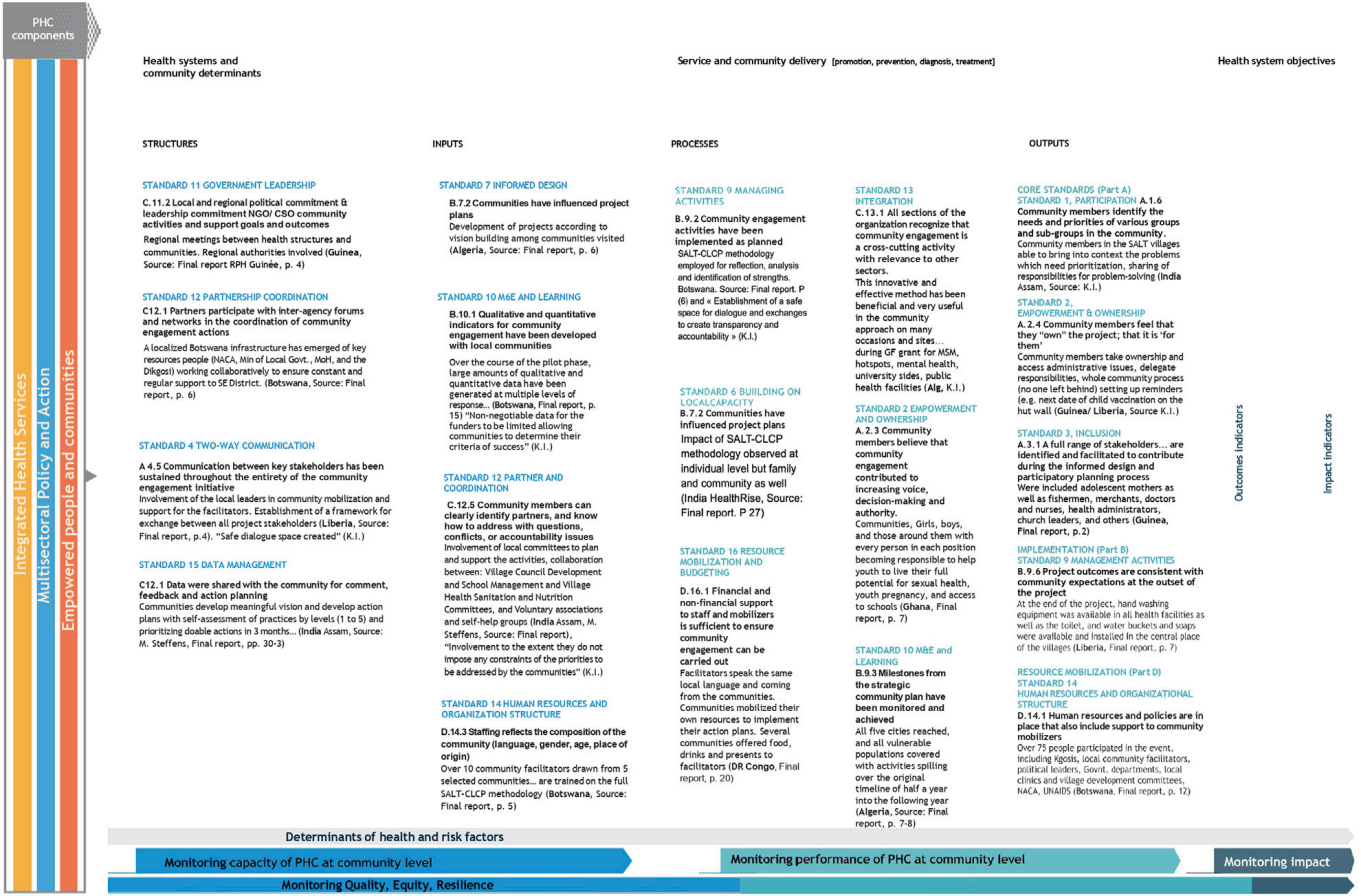
Comprehensive community-centered Monitoring and Evaluation (M&E) framework revisited—sample template with examples. Adapted from WHO/UNICEF guidance (framework) and UNICEF 2020 guidance (standards and indicators) with examples from 7 country projects, the Constellation.

We analyzed the evolution of existing M&E frameworks to integrate them into a comprehensive community-centered model, building on the latest WHO–UNICEF PHC measurement framework and UNICEF’s CE framework. Indicators were organized according to standard categories—structures, inputs, processes, and outputs—to facilitate CE outcome measurement. Selected indicators from the case studies were used as examples to illustrate these categories in the new Comprehensive Community-Centered M&E Framework Revisited–Sample Template with Examples from the case studies (Figure 1).

## Results

This section reviews international M&E frameworks, emphasizing their evolution toward community engagement (CE). It then draws on multi-country experiences from *The Constellation* to highlight lessons on CE and qualitative indicators. Finally, it proposes the development of a new comprehensive, community-centered M&E framework to better measure CE activities.

### A Chronological Overview of Community Engagement in International M&E Frameworks

Community frameworks emphasize indicators related to local activities—such as healthcare access and rights—aligned with the social determinants of health [25, 26]. Community health workers collect quantitative data on clients reached, facility access, and provider training, thereby playing a key role in achieving universal health coverage (UHC).

Over the past four decades, HIV/AIDS responses have illustrated how community involvement has strengthened public health responses and shaped international M&E frameworks. For example, HIV/AIDS M&E frameworks incorporated adaptable indicators that supported initiatives such as “Treat 3 Million by 2005” and the 95-95-95 targets [5, 27]. However, international M&E frameworks have tended to undervalue CE indicators, prioritizing project performance metrics over long-term benefits. These frameworks typically focus on quantitative data organized within logic models (inputs, processes, outputs, outcomes, impact). They emphasize national achievements, gaps, and funding priorities but often fail to effectively measure CE [2, 28]. Moreover, international agencies have not consistently aligned efforts to develop comprehensive CE indicator guidelines [28].

Table 1 summarizes several key recent contributions from four international agencies: UNICEF (2020), WHO (2022), UNAIDS (2021, 2023), and the Global Fund (2023) [4, 6, 7, 10, 23, 37].

**TABLE 1:** Analysis of international agencies’ contributions to monitoring and evaluation (M&E) frameworks (Switzerland, 2024).

UNICEF
UNICEF defines “Community” as “the minimum social unit locally relevant just above the level of the household (neighborhood, canton, precinct, parish, town, village).” “Community Engagement” is considered fundamental for collaboration with traditional societies, communities, civil society, governments, and opinion groups
UNICEF’s CE guidance outlines 16 detailed, multisectoral indicators—beyond health—to support PHC for SDG3 targets, applicable by civil society, implementers, and governments [4]. these indicators span four key areas: core CE, implementation, coordination and integration, and resource mobilization. They encourage quantification using a likert scale (1–5), drawing from post–Alma-Ata evidence [29]
WHO
At the 2018 Astana conference, the WHO director-general called for the integration of community health worker programs into national, regional, and local structures to achieve sustainable success in planning and implementing PHC [2, 30]
WHO’s 2022 HIV guidelines illustrate the gap between international M&E indicators and CE, focusing more on global policy than on community-level M&E [31]. WHO’s UHC compendium limits data collection tools to facility and population-based surveys, excluding qualitative community-level tools [32]. Only two of the six strategic objectives in WHO’s 2025–2028 general programme of work relate to community engagement [33]
UNICEF and WHO jointly published a measurement framework for PHC in 2022, recognizing primary care’s focus on equitable access to services while emphasizing service delivery, structures, inputs, and systemic functions [3, 22]. While primarily service-delivery focused, it incorporates CE structures and community linkages
UNAIDS
UNAIDS identifies Community-Led Monitoring (CLM) as a key accountability mechanism [5, 23, 34]. In its latest strategic plans and normative guidance, international agencies and civil society jointly document the importance of CLM [35]
The Global Fund
The Community Systems Strengthening (CSS) framework (2014) developed country-specific CE indicators, recognizing the value of qualitative methodologies (e.g., action research, operational research, focus groups, case stories, narratives, key informant interviews) to capture the experiences of vulnerable groups [36]. The current strategy prioritizes integrated, data-driven, community-centered services for national health program objectives [7]. It identifies CE as essential to achieving key performance Indicators, three of which (S4, S5, and C1) directly relate to community systems. Recent global fund efforts emphasize meaningful integration of community components into national and international M&E frameworks and dashboards, advocating for a holistic, actionable measurement framework for CE [36].

Each agency’s approach still presents challenges to creating a comprehensive M&E framework with a CE lens.

While UNICEF guidance identifies an essential package of CE indicators, they are not structured within standard M&E frameworks, and no other agencies have adopted them [4]. Despite WHO’s recognition of CE in PHC strategies, its 2022–2030 HIV strategy lacks dedicated CE indicators [6]. “Engaging empowered communities” is one of five strategic directions, yet only three of fifty-five actions (Actions 33, 34, and 55) address community-led and community health worker implementation. PHC and HIV guidance remains limited, omitting cross-cutting CLM for other infectious diseases, NCDs, or neglected tropical diseases [9].

The joint WHO–UNICEF guidance on PHC measurement frameworks acknowledges structure, input, and process indicators but remains more tailored to member states than to civil society organizations [22]. While UNAIDS has advanced CLM advocacy, it still lacks standardized community indicators and clear frameworks for measuring CE at the community level. CLM guidance (2021) presents quantitative and qualitative program results to document good practices, challenges, and recommendations but does not elaborate on specific community indicators [37]. The Indicator Matrix includes only two community-led response indicators out of forty-five (4.1.1 and 4.2.1) [38].

The Global Fund’s CSS guidance (2023) highlights the importance of CLM in addressing national challenges in community-led research, advocacy, capacity building, coordination, and rights-based, data-driven decision-making but overlooks CE indicators and measurement [39]. CLM is one of four CSS interventions prioritized for country-level funding, yet only one coverage indicator tracks CSS activity globally. As recent evaluations show, CE has often been treated as a “check-the-box” activity rather than a meaningful process, with no consistent measurement indicators [40].

A global understanding of which community indicators can effectively measure progress at the local level remains lacking. A Key Performance Indicator (KPI) capturing community contributions to program results is essential. Endorsing qualitative measures at the board and national levels could strengthen an enabling environment for CE and community-led responses globally.

Overall, the absence of consolidated guidance for community action remains a critical challenge. These gaps highlight the need for a comprehensive, community-centered M&E framework that integrates CE indicators. Building on recent advancements, our findings propose such a framework, incorporating lessons from an NGO aligned with international and community M&E approaches.

### Case Studies: Multi-Country Project Experiences

This section examines cross-national case studies illustrating how policymakers balance domestic needs with global primary care challenges [3]. NGOs play a crucial role in bridging gaps between international and community-level M&E frameworks. The Movement for Community-Led Development—a network of over 2,000 local organizations—developed a participatory tool to measure CE, inspiring international CLM efforts [41]. CE has also proven valuable in improving health outcomes [35, 42].

We reviewed case studies from seven countries over the past decade, based on *The Constellation’s* experience as an international NGO. *The Constellation* defines Community Engagement as a group’s capacity to unite and solve challenges, consistent with UNICEF’s definition. It employs the SALT–CLCP methodology, which merges an appreciative approach (SALT: Support, Appreciate, Learn, Transfer) with a practical process (CLCP: Community Life Competence Process). This approach offers accessible, non-technical tools applicable across educational and social levels and remains relevant and actionable after project completion. It also fosters sustainable participation with minimal external aid, though further details fall beyond this paper’s scope [42, 43].

Supplementary Material 1 summarizes lessons from these projects (2012–2022), covering populations from 100,000 to 3 million and underscoring CE’s role in achieving outcomes and impact.

*The Constellation*, in collaboration with the Public Health Foundation of India, evaluated the SALT approach using a cluster-randomized design to promote community ownership of immunization challenges [44]. This design enabled analysis across higher-level indicators (outcomes and impact) as well as categories directly relevant to CE (inputs, processes, and outputs). The HealthRise Transition Program employed an M&E framework combining data analysis with qualitative interviews [45]. Both initiatives inspired the development of the comprehensive, community-centered M&E system presented below. Indicators were co-developed collaboratively between project managers and community members from all seven countries. To ensure relevance and comparability, these indicators were retrospectively analyzed alongside those from UNICEF, WHO, UNAIDS, and the Global Fund.

Unlike traditional M&E frameworks that prioritize outcome and impact indicators, this approach emphasizes structural, input, process, and output indicators to better understand community dynamics and enable adaptive interventions [17]. From the comparative analysis of the case studies (Supplementary Material 1), we selected indicators that best captured community-level progress and change.

Our case studies reveal key gaps in measuring CE outcomes, highlighting three main constraints (as summarized in Supplementary Material 1):

- Short timeframes and limited funding: Many projects lasted only about 1 year (Columns 1 and 5), restricting the ability to capture CE results. Longer-term projects in the DRC and India showed stronger measurement.
- Evaluation design limitations: Quasi-experimental or pre-experimental one-group pretest–posttest designs (Column 3) are cost-effective but limited [46]. Alternative methods, such as outcome harvesting, may yield deeper insights.
- Challenges with qualitative methods: Storytelling, narrative inquiry, and key informant interviews provide rich evidence but require adaptable coding systems and well-defined variables.

Formative evaluations using qualitative methods helped identify a Most Significant Change (MSC) in each country. This contrasts with performance management evaluations, such as results-based M&E by the Global Fund and WHO, which rely primarily on quantitative indicators. Confidentiality concerns may also arise when assessing MSC among vulnerable populations [47].

Table 2 presents MSCs and selected quotes from engaged communities in three countries, collected through storytelling and focus group discussions. As early HIV/AIDS commentary observed, when monitoring data are reported without stories about real people, “the numbers do not take a life of their own.” [28] Findings indicate that fostering CE readiness supports program success and sustainability. Appreciative dialogue, cohesion, and recognition of community strengths enhance ownership, while diverse dialogue broadens understanding of local challenges. Final evaluations identified the most significant changes (MSC) through personal narratives.

**TABLE 2:** A few gems collected as Most Significant Change (MSC), from story-telling and focus group discussions (Democratic Republic of the Congo/Guinea/Liberia/India, 2017–2023).

Democratic Republic of Congo (final report, November 2023)
MSC: Behavioral change to accept routine and mass vaccination (National EPI program/UNICEF/CDC Atlanta) among religious leaders and their followers, among traditional health workers, some local populations (ex: Pygmies) and other individuals hostile to the use of health services
Story: (Translation from French): “We have known the vaccine: in the schools, market places, and then the vaccination campaigns a few times. This has provoked some curiosity from the community, with some rejections among those and queries among others. With the SALT approach, the community evaluated its competencies in full vaccination. This led to the implementation of an action plan which emphasized respecting the vaccination calendar, for pregnant women as well as for children. Today the women of KABEYA MAY respect the vaccination calendar and their children are fully vaccinated”. (from the village chief’s wife and community relay, p. 15)
Guinea and liberia (final report, August 2017)
MSC: Restoration of confidence in the health systems leading to increased use of health services, and maternal and child health services with ANC visits and births. (Outputs)
Story: Evidence from the project illustrated renewed dialogue and ownership of Health services and on other development indicators (waste management, sanitation, prevention of transmitted diseases)
India (Udaipur and Shimla) (Health Rise transition grant project report, October 2020)
MSC: Favorable changes and improvements in the lifestyle behaviors, self-care practices (outcomes), village sanitation, dietary habits, organic farming, physical activity, clinical control among patients (diabetics and hypertensive) and metabolic control (impact) after the implementation of the program in the intervention arms
Story: “These meetings can still be held in the village… Everybody from the village gathers and if there are any small issues, we resolve them. If somebody does not know about a particular disease and another person has knowledge about it, we share the information. In this way, we help each other out… One man got hospitalized and was given a lot of medicines for de-addiction (alcohol) and after that he quits. After listening to his plight, 4-5 others stopped drinking alcohol. They heard this story in SALT meetings … Many changes have happened in the village. People were not caring about cleanliness and health initially. Now people have understood the importance of handwashing, good food habits, drinking water after boiling and keeping their surroundings neat and clean.” (patient, Shimla, p. 34)

### Towards a Comprehensive Community-Centered M&E Framework

To advance CE within the 2030 UHC agenda, we propose integrating community indicators into existing M&E frameworks, emphasizing qualitative data collection and community participation. Rather than maintaining separate systems, we consolidate community indicators into a single unified standard framework, linking and revisiting existing ones. Guidelines and frameworks alone are insufficient—bridging the gap between international and community M&E indicators is essential.

As illustrated in Figure 1, our proposed framework integrates UNICEF’s CE indicators within WHO’s four-category model, selecting relevant standards and indicators identified by UNICEF and its partners. UNICEF’s CE indicators were primarily designed for NGOs, civil society organizations, and implementers to support health service delivery models; our comprehensive framework expands upon these to include a selection of UNICEF’s 16 multisectoral quality standards and indicators based on examples drawn from country-level data and evaluation reports.

This framework is a comprehensive template constructed from standard M&E structures. Using the WHO PHC framework, Figure 1 demonstrates how to measure the effectiveness of community engagement and service delivery. “Structures” and “Inputs” (left side) monitor community-level PHC capacity, while “Processes” and “Outputs” (right side) measure community-level PHC performance. Outputs represent the highest-level indicators and are crucial for assessing outcomes and impact at the community level. The framework aligns UNICEF standards with corresponding indicator categories, drawing examples from *The Constellation’s* case studies.

Our framework harmonizes existing international M&E frameworks with CE indicators, drawing on UNICEF, WHO, UNAIDS, and partners’ recommendations. Inspired by recent WHO and UNICEF frameworks and by community-focused M&E work for NCDs in India, contextualized through *The Constellation’s* country-specific indicators (above and Supplementary Material 1), the comprehensive framework serves as a flexible, adaptable tool for national stakeholders. Informed by contemporary evaluation tools and qualitative methodologies, it supports measurement and acceleration of community responses across infectious diseases, NCDs, and rare diseases under a PHC approach [48].

The overarching goal is to integrate this new framework into routine data collection for CE at both local and global levels.

In conclusion, our findings build upon UNICEF’s 2020 CE indicators and WHO’s latest model, emphasizing the structures, inputs, and processes that generate substantial outputs and contribute to broader national outcomes and impact [4, 22]. We next discuss how this new framework can be adapted for implementation in the near future.

## Discussion

This paper presents a chronological review of the evidence and identifies existing gaps in measuring community engagement (CE) within evolving international monitoring and evaluation (M&E) frameworks. It also compiles qualitative methods and indicators gathered over several years through a multi-country initiative (The Constellation). The central recommendation is the development of a comprehensive, community-centered M&E framework—structured as an adaptable template for diverse projects and partners.

Western aid agencies have traditionally emphasized selective interventions; however, primary healthcare (PHC) and CE remain essential for achieving universal health coverage (UHC) and the Sustainable Development Goals (SDGs). Sustained progress in the Global South continues to depend on community participation [2]. CE is vital for attaining public health and development outcomes. While strategic guidance and funding are fundamental, effective M&E is central to achieving meaningful CE [1].

Drawing on the authors’ experience in developing M&E frameworks, advising countries on program implementation, and reviewing the literature, this section addresses five key areas: 1) adaptation of the new M&E framework, 2) research, 3) digital innovation, 4) resource mobilization, and 5) recommended actions. Each area targets stakeholders involved in health system strengthening and warrants special attention for the successful adaptation and implementation of the proposed comprehensive framework.

### Adaptation of a Comprehensive Community-Centered M&E Framework Revisited

Since the 1990s, CE methodologies have evolved considerably [30, 36]. Yet, program indicators still tend to prioritize outcomes and impact, often neglecting input-to-output indicators—despite attempts to consolidate guidelines under unified structures [49]. International M&E frameworks frequently overlook qualitative community approaches due to insufficient evidence and the prioritization of high-level quantitative indicators for global reporting. Although the Millennium Development Goals and SDG strategies acknowledge local communities, standardized top-down indicators often fail to capture the bottom-up measures preferred by these communities.

Our findings highlight the importance of community-based M&E methodologies that emphasize qualitative indicators. Rigid international standards rarely reflect community-driven health improvements. Effective feedback mechanisms—such as the use of Most Significant Change (MSC)—allow communities to interpret M&E results for reflection, planning, and action [50]. While international agencies have begun integrating CE measurement, efforts remain fragmented and insufficiently aligned with emerging monitoring frameworks. Moreover, qualitative data collection methods and tools are often underused [49].

The flexible evaluation framework (Figure 1) aligns with UNICEF indicators and the Global Fund’s 2023–2028 strategy, facilitating integration of CE indicators [7]. This comprehensive framework represents a first step toward a community-centered M&E system. Case studies illustrate various indicator types corresponding to UNICEF’s four core standard categories. Further adaptation by global agencies is needed to align with international M&E standards and broader community-led development movements (see Annex: Participatory CLD Assessment Tool) [41].

International agencies increasingly recognize that participatory CE enhances PHC, social services, and all SDGs. The synthesis following Table 1 highlighted key challenges in existing M&E frameworks and required adaptations. Since 2020, several efforts have been made to measure community-level results both qualitatively and quantitatively; however, persistent challenges include:

- Lack of international consensus: No standardized CE M&E framework currently exists, resulting in fragmented approaches.
- Absence of structured CE indicators: Existing indicators are not systematically categorized within standard frameworks.
- Limited use of qualitative methods: Social science methodologies remain underutilized.

The proposed framework is adaptable to national and local program cycles, particularly during the planning phase to define methods, tools, and indicators early. It empowers communities and program staff to select contextually relevant indicators. As shown in Figure 1, several generic CE indicator types can illustrate progress while allowing communities flexibility to choose indicators aligned with UNICEF CE standards and local priorities.

This flexible global framework supports the co-development of project indicators, enabling effective community M&E, inclusion of community data in broader datasets, empowerment of stakeholders, and alignment with funder expectations.

### Research

Research on community-based metrics remains limited, though two recent studies offer promising directions. PHC research across four Latin American countries found no universally accepted operational definition for PHC—highlighting the need to address both essential services and intersectoral actions targeting distal, harder-to-measure determinants of health [51]. In three sub-Saharan African countries, a Primary Care Assessment Tool using exit interviews was developed to evaluate service quality and core primary care functions but excluded teamwork and community orientation [52].

Recognizing that current indicator mapping may not fully align with UNICEF’s core standards, the proposed template offers a promising area for further research and adaptation. We recommend that agencies and partners continue refining and testing the template (Figure 1) through multi-country studies.

Since the early 2000s, logical frameworks—widely used in development and government strategies—have historically neglected CE [28]. These frameworks typically measure inputs, outputs, outcomes, and impacts through quantitative trends at global or local scales [25]. Their static, linear models are ill-suited to capturing systemic change, unlike recent adaptive, network-based models [50, 53].

Further research is needed to identify relevant key performance indicators (KPIs) for seamless integration into national M&E systems, ensuring frameworks are tailored to community needs and aligned with national public health standards. Mixed-method designs such as outcome harvesting can yield deeper insights into CE processes than traditional outcome-based evaluations [44, 53]. These approaches are particularly useful for large-scale initiatives where aggregated indicators may obscure local dynamics.

Initiatives such as the Second Generation HIV Surveillance and COVID-19 studies in Switzerland and France demonstrate that behavioral constructs—like beliefs and information-seeking behaviors—can be effectively measured [54–56]. However, cross-sectional designs limit the assessment of cultural change over time [57, 58]. Our findings support the inclusion of behavioral and programmatic indicators (including KPIs) in CE monitoring frameworks.

Qualitative methods such as storytelling and focus groups add contextual depth, particularly within global health metrics [59, 60]. These “soft” data sources enhance understanding of complex realities and support nuanced evaluations [28]. Rigorous qualitative approaches, including triangulation and interpretive analysis, have proven valuable in health and environmental impact assessments [61, 62].

Participatory methods, quasi-experimental designs, and the MSC technique (Table 2) can effectively bridge quantitative and qualitative data, as demonstrated by large-scale CE projects in India conducted by *The Constellation* over extended timeframes [45, 63]. We advocate for evaluation frameworks that capture both process and outcome dimensions, ensuring comprehensive assessments of CE initiatives [64].

### Digital Innovation

Digital innovation strengthens community-centered M&E for PHC by leveraging technology to improve disease prevention, health promotion, and service delivery [36, 65]. The COVID-19 pandemic accelerated digital transformation, enabling countries to monitor services, follow-ups, and community engagement via dashboards and data visualization tools. Nonetheless, adoption remains limited in low- and middle-income countries, particularly among community health workers [66]. Examples from Botswana and the Democratic Republic of Congo show that community dashboards can enhance multilingual communication and qualitative data analysis [67]. Techniques such as longitudinal coding can streamline evaluations and integrate quantitative and qualitative data, increasing efficiency and insight [53]. However, strong safeguards for cybersecurity, human rights, and privacy are essential as digital systems become embedded in health frameworks [68].

### Resource Mobilization

Resource mobilization is critical for implementing any M&E framework. The WHO estimates that half of the world’s population lacks access to essential health services, underscoring the need for greater PHC investment [69]. CE initiatives benefit when leaders and funders prioritize PHC expenditures beyond national budgets [70]. While community programs often mobilize local human and material resources, additional national and international funding is usually necessary.

### Recommended Actions

We recommend adopting the proposed community-centered M&E framework across NGOs, civil society, and implementing agencies. Priority actions (Table 3) target five key stakeholder groups in global health, promoting policy reforms to strengthen community development systems. Large-scale research and dedicated funding are essential to validate this framework in line with UNICEF and WHO guidance, advancing CE measurement and fostering paradigm shifts in policy and practice.

**TABLE 3:** Recommended priority actions for specific audiences (Switzerland, 2025).

For countries • Include CE and M&E as a core strategy in development plans and budgets • Create mechanisms to integrate and finance lessons learnt locally from CE towards sustainable results • Engage key stakeholders beyond health (finance, agriculture, environment, education, labor)
For national and international NGOs • Increase funding for community M&E systems (human resources, digital health, training) • Support CE research, development and implementation • Strengthen staff capacities for appropriate CE
For international agencies and foundations • Regularly update the comprehensive M&E framework revisited, with indicators (such as key performance Indicators for community participation), providing guidance, illustrations, and training materials • Align the agenda and efforts between agencies and partners and provide technical assistance • Increase funding for community M&E with particular attention to challenging operating environments • Facilitate collection, discussion and use of CLM lessons learnt at local, national and global levels • Support NGO interventions strengthening CE and SDGs
For academic institutions • Develop training modules (including affordable MOOCs) on CLM and innovation • Provide appropriate thought leadership to international agencies, NGOs and private sector partners implementing CE programs, for example, in the social sciences
For the private sector • Engage communities in public-private partnerships for equitable non-exploitative research and development • Collaborate with academic institutions to facilitate mutually beneficial CE research • Support international agency efforts in CE program implementation

### Limitations

Contextual constraints limited the application of a full PRISMA checklist for meta-analysis. Further research should refine qualitative attribution methods and integrate CE indicators into evaluation frameworks. Addressing social determinants of health—such as poverty and access to care—requires stronger evidence on the roles and responses of community systems [34].

Historical health system developments highlight the effectiveness of bottom-up interventions compared with top-down community expansion. As countries resume post-COVID-19 UHC efforts, expanding initiatives with communities, rather than for them, remains a significant challenge.

Global initiatives such as the African and U.S. Centers for Disease Control and Prevention’s event-based surveillance framework and UNAIDS–WHO’s Second Generation HIV/AIDS Surveillance demonstrate the value of standardized indicator development [71, 72]. Further research is needed to balance strategic funding frameworks with community-based indicators. Reviews of European HIV/AIDS prevention policies in the late 1990s emphasized the importance of structured multi-country evaluations [73]. More recently, the European & Developing Countries Clinical Trials Partnership in Africa has accelerated the evaluation of diagnostics, treatments, and vaccines for infectious diseases [74]. Ensuring equitable benefit-sharing and shared responsibility in these collaborations remains essential.

In summary, this paper advocates for a community-centered M&E framework that integrates qualitative and quantitative methods, promotes adaptation across settings, leverages digital innovation, ensures sustainable resource mobilization, and supports actionable recommendations for global health stakeholders.

### Conclusion

The WHO has designated the final decade of the SDGs as “a decade for disease elimination” [75]. As 2030 approaches, prioritizing marginalized subpopulations—whether affected by infectious diseases, NCDs, or neglected tropical diseases—remains essential to accelerating progress. However, early evidence suggests that international agencies have historically underemphasized measuring results to advance CE, despite promoting it in their global strategies.

Both international and national NGOs have developed their own CE metrics, complementing those of international agencies. Recently, international organizations have intensified their focus on CE measurement through CLM, while the African region has demonstrated the value of adaptable M&E frameworks for pandemic preparedness [16]. These developments underscore the need for subnational assessments tailored to context-specific needs.

Fifty years after the PHC declaration, integrating a comprehensive, community-centered M&E framework remains critical. Strengthening these frameworks will enhance CE evaluation and participation, requiring sustained commitment from policymakers and program implementers.

Development actors must update and apply these frameworks, invest in the social sciences—particularly qualitative methods—improve evaluation tools, and expand capacity-building efforts. Applied research should integrate these approaches throughout all stages of disease program cycles.

This publication seeks to align international M&E frameworks with current CE initiatives, incorporating national examples to develop a comprehensive, community-centered model that advances policy and practice in CE and PHC.
