# Supplementary material for: Accelerating Community Engagement: Measuring Results
Source: Int J Public Health. 2025 Dec 16;70:1608499. doi: 10.3389/ijph.2025.1608499 (PMC12747999; doi:10.3389/ijph.2025.1608499)
Supplement: Supplementary file 1 [file DataSheet2.pdf]

Supplementary Material 2: Summary Analysis of the Table

Data sources included final evaluation reports and interviews with key informants at national or international levels. At the community level, multisectoral activities involved community leaders, local NGOs, civil society and Ministry of Health representatives. Other organizations -such as research institutes, bilateral agencies, international organizations and foundations- participated for periods ranging from six months to one year (or longer in India). (Column 1)

All projects responded to initial requests addressing public health activities for infectious diseases (e.g. HIV/AIDS, COVID-19, immunization, malaria, diarrhea, infection prevention and control and hygiene, and reproductive health) and NCDs (e.g. hypertension and diabetes), targeting young people, vulnerable populations and broader community development. (Column 2)

Most evaluations employed pre-experimental designs (‘one-shot case study’), with a pretest-posttest design used in DR Congo to assess individual and community changes. Two projects in India adopted quasi-experimental designs with extended timelines to measure impact.[54] Community programs were frequently evaluated through participatory formative methods that incorporate narratives and insights from community members and program managers. [55] These programs focused on process and output indicators derived from qualitative M&E data, rather than solely on quantitative outcome and impact measures typically favored by donors such as the GF and bilateral funders. (Column 3)

All projects primarily utilized qualitative methods - including key informants, story-capturing, in-depth or group interviews, and self-assessments - to gather CE indicators. In addition, focus groups served as valuable tools for trained facilitators to collect in-depth insights on specific topics. (Column 4)

Because Health Information Systems often cannot report higher-level outcomes within short timeframes, outcome harvesting was used alongside impact evaluations to collect additional qualitative data. This approach engaged informants to retrospectively review processes, outputs and outcomes. While outcome and impact evaluations were typically conducted sequentially, a mixed-methods evaluation was piloted to integrate them. In India, two reports based on quasi-experimental study designs sought to demonstrate the causal effects of the SALT-CLCP methodology over three to five years. (Column 5)
