# Supplementary material for: Accelerating Community Engagement: Measuring Results
Source: Int J Public Health. 2025 Dec 16;70:1608499. doi: 10.3389/ijph.2025.1608499 (PMC12747999; doi:10.3389/ijph.2025.1608499)
Supplement: Supplementary file 2 [file DataSheet1.pdf]

Supplementary Material 1. Summary of The Constellation Projects in 7 Countries

Sources: final country or evaluation (in India) reports and key informant interviews

*MSC: Most Significant Change reported separately with examples in Box 1

**COUNTRIES**<br>Project (name)<br>Catchment area<br>Period<br>Partners<br>(in addition to *The Constellation*)	**Infectious/ NCDs/ Others**<br>(in addition to community engagement) with **Priorities**	**Evaluation Designs**<br>(in addition to cross-cutting strength-based SALT-CLCP methodology) and **MSC***	**Qualitative Methods**	**Evidence of Outcomes/Impact**<br>(Structures, Inputs, Process, Outputs: ref. section 4. Discussion)
**ALGERIA**<br><br>"**Improving access to health care for migrants and vulnerable populations in Algeria**" Project<br><br>(population coverage: unknown)<br><br>Febr. - Sept. 2022 (1/2 year)<br><br>MoH, City authorities, International Office of Migration (IOM), local NGOs/ CSOs, Associations/ Volunteers (e.g.Red Crescent) working with people in socially vulnerable situations and migrant populations from 5 cities/ Wilayas (Alger, Annaba, Bejaïa, Tamanrasset and Oran)	HIV/AIDS<br><br>COVID-19<br><br>Most vulnerable groups (e.g.<br><br>PLHIV,<br><br>migrants, MSM, LGBTQ+, sex workers, mental health patients)	Pre-experimental design ("one-shot case study")<br><br>Limited because confidentiality of beneficiaries strictly applied<br><br>Key informants, group discussion at hotspots (virtual or in person), university sites, public health facilities	Story telling (testimonies) at an individual, community and societal level<br><br>Key informants	Impact documented through storytelling<br><br>HIV testing (Global Fund financed):<br><br>787 in 2022, and 897 in 2023<br><br>Detailed outcomes and outputs: not documented for confidentiality reasons

**COUNTRIES**<br>Project (name)<br>Catchment area<br>Period<br>Partners<br>(in addition to *The Constellation*)	**Infectious/ NCDs/ Others**<br>(in addition to community engagement) with **Priorities**	**Evaluation Designs**<br>(in addition to cross-cutting strength-based SALT-CLCP methodology) and **MSC***	**Qualitative Methods**	**Evidence of Outcomes/Impact**<br>(Structures, Inputs, Process, Outputs: ref. section 4. Discussion)
**BOTSWANA**<br><br>Project CATCH (17,264)<br><br>Southeast district, 85,014, including Jwaneng Township<br><br>June 2015-May 2016<br><br>MOH/NACA, UNAIDS Botsw., CDC Botsw., USAID/FHI 360	Infectious disease (HIV):<br><br>overcoming stigma<br><br>HIV testing	Pre-experimental design ("one-shot case study")<br><br>Self-Assessment<br><br>Participatory evaluation (External evaluation with community representatives' participation)<br><br>Outcome evaluation (individual and community changes)	Narrative inquiry<br><br>Story-capture through text and video<br><br>Interviews	Contribution to overall goal of reducing HIV incidence to Zero (Impact)<br><br>New infections, access to ART, PLHIV, Knowing HIV status with testing (Outcomes)<br><br>"Many people are testing for HIV these days. In one campaign, 37 people tested to know their HIV status. Community member Taung." (Source: Final report, Botswana, p. 2)<br><br>The challenge to measure higher level indicators at district level (baseline vs project accomplishments) in a short period was acknowledged with the need to identify a set of measurable community health data at district level.
**DR CONGO (DRC)**<br><br>(Source: final report only)<br><br>Project GCVC-Gestion Communautaire de la Vaccination Complète District sanitaire-Tanganyika district<br><br>June 2012-November 2013<br><br>2.762.995 inhabitants	Immunization coverage<br><br>Additional:<br><br>Exclusive breastfeeding, pregnant women or giving births accompanied by partners, civil registration (9-months after births),	Pre-experimental design ("one-group pretest-post-test design")	Narrative inquiry<br><br>Story-capture<br><br>Interviews<br><br>Health Information System data analysis	8.477 children of more than 12 months who accessed routine vaccination (Outcome)<br><br>Access and good use of services doubled from 25 to 50% in the areas covered, and the poor use of services in that category diminished from 32.5 to 5% (Output)

**COUNTRIES**<br>Project (name)<br>Catchment area<br>Period<br>Partners<br>(in addition to *The Constellation*)	**Infectious/ NCDs/ Others**<br>(in addition to community engagement) with **Priorities**	**Evaluation Designs**<br>(in addition to cross-cutting strength-based SALT-CLCP methodology) and **MSC***	**Qualitative Methods**	**Evidence of Outcomes/Impact**<br>(Structures, Inputs, Process, Outputs: ref. section 4. Discussion)
(400 communities in the 8 zones/ 40 areas)<br><br>National EPI program<br><br>CDC Atlanta, UNICEF	Impregnated bed nets, handwashing, schooling of children			
**GHANA** and multi-country (Ghana, Kenya, Cameroon, India, Indonesia)<br><br>Project Go-Girl Ghana (GGG)<br><br>(85,014, South East District)<br><br>2021-2022<br><br>The MoH and<br><br>Communities in Nyanyano and Buduburam	Youth to live their full potential for sexual health, youth pregnancy, and reduced dropout from school	Pre-experimental design ("one-shot case study")<br><br>Needs self-Assessment<br><br>Outcome evaluation (individual and community changes)	Narrative inquiry<br><br>Story-capture<br><br>Interviews	Not documented
**GUINEA** and **LIBERIA**<br><br>Project Régional Confiance (PRC) Project<br><br>8 communities and 4 health centers (Guinea) and 3 (Lib.) at the border<br><br>(population sizes, not informed)	Immunization coverage, Access to Repr. Health services, Diarrhea, Malaria, Infection Prevention and Control (IPC), Hygiene<br><br>Increased access and use	Pre-experimental design ("one-shot case study")<br><br>Needs self-Assessment<br><br>Communities' own assessments or monitoring own	Stakeholder/ community mapping<br><br>Narrative inquiry<br><br>Story-capture<br><br>Community satisfaction and health care	At the population level, evidence of behavior changes (Outcomes) by increase in access health facilities- ANC visits and general treatment (Outputs) in 16 communities (Communes) in both countries<br><br>In Guinea: the number of pregnant women coming to the last antenatal care (ANC) visit increased 57% over one year in

**COUNTRIES**<br>Project (name)<br>Catchment area<br>Period<br>Partners<br>(in addition to *The Constellation*)	**Infectious/ NCDs/ Others**<br>(in addition to community engagement) with **Priorities**	**Evaluation Designs**<br>(in addition to cross-cutting strength-based SALT-CLCP methodology) and **MSC***	**Qualitative Methods**	**Evidence of Outcomes/Impact**<br>(Structures, Inputs, Process, Outputs: ref. section 4. Discussion)
Sept. 2016-Aug. 2017 (post-Ebola)<br><br>Antenna Tinkisso (Guinea),<br><br>German Inst for Medical Mission/DIFÄM, CHAL, additional technical support with GiZ	of health services, and Ante-Natal Clinics (ANC)	development processes<br><br>Outcome evaluation (individual and community changes)	providers' surveys<br><br>Interviews<br><br>Observation (e.g. latrines, water buckets and soaps, refurbishing of health facilities)	3 of 4 communities served by 1 facility (from a total of 4 facilities)<br><br>The number of patients coming for general treatment increased between 44% and 1123% among the 4 facilities in all 8 communities over the same period.<br><br>In Liberia:the number of pregnant women coming to the last antenatal care (ANC) visit increased by 800% over one year, in 6 of 8 communities served by 3 facilities (Table 1, p.5). The number of patients coming for general treatment increased 300% among the 3 facilities in the same number of communities over the same period
**INDIA (Assam)**<br><br>The SALT Project in Assam<br><br>3 districts, Total: 3,088,014 inhabitants: Bongaigaon (738,804), Kamrup (1,517,542), and Udalguri (831,668)<br><br>3 years, participatory evaluation.	Increased access of the rural population to immunization services<br><br>But other issues identified by communities: school dropout, quality of education, recreation time of children,	Impact evaluation:<br><br>cluster randomized design with two groups (Two rounds of surveys conducted before and after the SALT-CLCP intervention)<br><br>And separately:	Consensus building (problem-solving , prioritization of doable activities)<br><br>In both evaluations use of:<br><br>Narrative inquiry<br><br>Story-capture through text and video	Increased utilization of immunization coverage. (Outcome)<br><br>Consistent high coverage over time. (Outcome)<br><br>DPT3 coverage significantly increased between National Family Health Survey 2015–16 (NFHS-4) and SALT baseline (June-Aug 2016); and remained at a high level until SALT endline (July-Sep 2018) in three districts: Bongaigaon 69.1% to 80.8% (78.8 at baseline);

**COUNTRIES**<br>Project (name)<br>Catchment area<br>Period<br>Partners<br>(in addition to *The Constellation*)	**Infectious/ NCDs/ Others**<br>(in addition to community engagement) with **Priorities**	**Evaluation Designs**<br>(in addition to cross-cutting strength-based SALT-CLCP methodology) and **MSC***	**Qualitative Methods**	**Evidence of Outcomes/Impact**<br>(Structures, Inputs, Process, Outputs: ref. section 4. Discussion)
Centre for North East Studies and Policy Research (C-NES) and the Voluntary Health Association of Assam (VHAA). Evaluations by the International Initiative for Impact Evaluation, informed by S. Pramanik et. al., and Outcome harvesting by Michael Steffens	nutrition, cleanliness<br><br>and sanitation, and water cleanliness	Outcome harvesting methodology evaluation	Interviews<br><br>Self-Assessment before and after the SALT- CLCP<br><br>Intervention (Outcome harvesting)	Kamrup rural 54.8% to 80.4% (85.3% at baseline); and Udalguri 80.5 to 88.2% (88.2% at baseline).<br><br>Observable behavioral changes (Outcome)<br><br>Improvements in practices of regular immunization were reflected by the changes in practices in the villages: “taking our children for immunization timely” – improved regularity of immunization from “We do it sometimes” (2017-18 Self-Assessment) to “We do it regularly” (2018-19 Self-Assessment). (in Outcome harvesting, Table 3: Self-Assessment before and after the SALT – CLCP intervention, p. 12).

**COUNTRIES**<br>Project (name)<br>Catchment area<br>Period<br>Partners<br>(in addition to *The Constellation*)	**Infectious/ NCDs/ Others**<br>(in addition to community engagement) with **Priorities**	**Evaluation Designs**<br>(in addition to cross-cutting strength-based SALT-CLCP methodology) and **MSC***	**Qualitative Methods**	**Evidence of Outcomes/Impact**<br>(Structures, Inputs, Process, Outputs: ref. section 4. Discussion)
**INDIA (Udaipur and Shimla)**<br><br>The HealthRise Transition grant Project.<br><br>Population sizes (2011): Shimla District (814,010),Udaipur District (3,068,420). 6 year project (during COVID-19: 2015-2020).<br><br>Funded by Medtronic Foundation. Implementation: MAMTA- Health Institute for Mother and Child, Catholic Health Association of India (CHAI), Ramaiah International Centre for Public Health Innovations (RICPHI)	Diabetics and hypertensive patients	Impact evaluation: longitudinal four arm quasi-experimental study design<br><br>Baseline and 3 rounds of surveys encompassing disease condition, health seeking<br><br>behavior and lifestyle habits	Qualitative interviews with quotes<br><br>In-depth interviews<br><br>Group interviews<br><br>Stories of change through text<br><br>Manual coding	Improved clinical control among patients (diabetics and hypertensive) with controlled blood pressure as well as blood sugar levels/ metabolic control of Diabetes and Hypertension (Impact) (Controlled disease status of participants across the study arms, Shimla and Udaipur, Fig. 11, p. 21)<br><br>Favorable changes in the lifestyle behaviors, self-care practices in physical activity levels, in dietary practices, tobacco and alcohol consumptions, medication adherence (Outcome) (Summary and Conclusion, p. 30)
